# Apolipoprotein variations across APOE genotypes in young and elderly patients with coronary heart disease

**DOI:** 10.1042/BSR20260258

**Published:** 2026-06-12

**Authors:** Yuxuan Zhang, Siwei Li, Shijie Xu, Rui Zhao, Fang Zheng, Junfang Wu

**Affiliations:** 1 Division of Cardiology, Department of Internal Medicine, Tongji Hospital, Tongji Medical College, Huazhong University of Science and Technology, Wuhan, China; 2 Hubei Key Laboratory of Genetics and Molecular Mechanisms of Cardiological Disorders, Wuhan 430000, China; 3 Center for Gene Diagnosis, Department of Laboratory Medicine, Zhongnan Hospital of Wuhan University, Wuhan 430071, China

**Keywords:** APOE genotypes, APOE isoform-specific peptides, ApoL1, apolipoproteins, coronary heart disease, Liquid chromatography triple quadrupole mass spectrometry

## Abstract

Both the Apolipoprotein E (ApoE) genotype and apolipoprotein (Apo) profiling are closely associated with the risk of coronary heart disease (CHD). However, it remains unclear whether APOE genotypes modulate the distribution of Apos and whether such regulation differs between young and elderly CHD patients. The present study aims to investigate the effects of APOE genotypes on the levels of plasma Apos in both young and elderly CHD patients. Two datasets were analyzed. Dataset 1 included 293 CHD patients with APOE genotyping. The quantification of 4 APOE isoform-specific peptides and 16 Apos was simultaneously measured in plasma using liquid chromatography-mass spectrometry. Dataset 2 comprised 3821 CHD participants from UK Biobank, for whom Apo levels were obtained through Olink proteomics. Selected Apos identified in dataset 1 were independently validated in dataset 2. We developed a robust method for simultaneous quantification of APOE isoforms and Apos in human plasma. APOE genotype results from liquid chromatography-mass spectrometry were fully consistent with those from the TaqMan assay in all patients. Among the measured Apos, the levels of ApoL1 were significantly decreased in elderly CHD patients, which were independently validated in the UK Biobank cohort, particularly in those carrying APOΕ ε3 and ε4 alleles. Mediation analysis revealed that ApoL1 statistically mediated the association between age and CHD status. Our results suggested that APOE polymorphisms affect the plasma Apo profiles and that ApoL1 is associated with older CHD patients carrying the APOE ε3 and ε4 genotypes.

## Introduction

Coronary heart disease (CHD) is a globally prevalent chronic disease, commonly seen in elderly individuals over 45, but it has been gradually increasing in younger populations as well [1]. According to the World Health Organization, the incidence of CHD in younger individuals has risen by 50% over the past decade due to their unhealthy lifestyles and lack of exercise. From the PROSPECT study, Ruiz-García revealed that heavier plaques in elderly CHD patients were predominantly composed of necrotic cores and dense calcification, whereas plaques in younger CHD patients were mainly characterized by fibrotic components [2]. Despite these insights, research directly comparing CHD in young versus elderly populations remains limited, posing challenges to precise prevention strategies targeted at CHD at different stages.

Apolipoproteins (Apos) are essential components of lipoproteins, playing a critical role in lipid transport and maintaining lipoprotein structural stability. Previous studies have demonstrated a close correlation between Apos and the risk of CHD. A global study across 52 countries identified the ApoB/ApoA1 ratio as a key risk factor for myocardial infarction [3]. Additionally, other Apos including ApoC1, C2, and C3 have been closely linked to CHD risk, with ApoC1 levels independently predicting early atherosclerosis in healthy middle-aged men [4]. The PROCARDIS study further highlighted the association between Apo profiles and residual risk of CHD [5]. Moreover, Mendelian randomization, epidemiological, and prospective studies have established lipoprotein(a) [Lp(a)] as an independent risk factor for atherosclerosis [6]. Clinical interventions targeting LDL-C reduction through PCSK9 inhibitors [7] have already been approved, while therapies targeting ApoA1 [8] and ApoC3 [9] are currently in clinical trials. Notably, CSL112, an injectable formulation based on ApoA1 [10], has shown potential in reducing recurrent cardiovascular events among high-risk CHD patients. These findings highlight both the prognostic value of Apos in cardiovascular disease and their clinical translational potential as therapeutic targets.

The APOE (Apolipoprotein E) genotype is also important in the development of CHD. The three major alleles (ε2/ε3/ε4) differ in their influence on lipoprotein metabolism efficiency and atherosclerosis progression [11]. APOE genotyping plays a central role in regulating multiple key aspects of Apo metabolism by affecting the structure and function of ApoE protein, thus significantly affecting the lipid profile and contributing to cardiovascular disease risk. For example, genome-wide association studies have reported that APOE impairs the production but not the catabolism of Lp(a) [12]. Moriarty et al. suggested that differences in the binding affinity of ApoE isoforms for lipoprotein clearance receptors may modulate Lp(a) catabolism, indicating potential competition between Lp(a) and ApoE for receptor-mediated clearance [13]. Additionally, the APOE ε4 genotype is associated with increased LDL-containing ApoB particle numbers and promoting the atherosclerosis [14]. Gerritsen et al. found that the ε2 genotype may lead to the accumulation of residual particles and elevated ApoC-III, which inhibit lipoprotein lipase activity and exacerbate hypertriglyceridemia [15]. However, the impact of different APOE genotypes on Apos in elder CHD patients remains poorly understood.

Mass spectrometry-based proteomics is an accurate and reliable method for quantifying Apos, including Lp(a) [16]. Recently, Olink-based proteomics has also been able to provide relative quantification information for several Apos. With the exploration of the UK Biobank (UKB) Olink proteomics platform, plasma proteomics have enabled large-scale analysis of nearly 3000 plasma proteins across 406 common diseases in over 53,000 participants, providing the most comprehensive protein landscape to date [17]. The combined application of Olink proteomics and whole-exome sequencing data on the UKB platform would provide direct insights into the impact of APOE genotypes on Apo levels in CHD patients.

In the present study, we utilized two datasets. Dataset 1 included 293 CHD patients across both young and elderly populations, in which we quantified ApoE isoform-specific peptides and 16 additional Apos using liquid chromatography triple quadrupole mass spectrometers (LC-QQQ-MS) to examine the impact of different APOE genotypes on Apo expression in elderly CHD patients. Dataset 2 served as an independent validation cohort that comprised CHD participants from the UKB, where APOE genotypes were obtained through whole-exome sequencing data and Apo levels were measured via the Olink proteomics platform. Our study aims to assess the effects of APOE genotypes on Apo profiles in CHD patients across different ages.

## Materials and methods

### Study participants and blood sample collection in dataset 1

A total of 293 CHD patients from Zhongnan Hospital of Wuhan University between June 2024 and October 2024 were enrolled in the present study, comprising 75 patients aged ≤45 years and 218 patients >45 years. The diagnosis of CHD was confirmed by experienced clinicians in accordance with the American College of Cardiology and American Heart Association guidelines [18], integrating clinical symptoms, physical examination, laboratory tests, and coronary angiography or other imaging evidences.

Remaining blood samples from clinical laboratory tests of patients who underwent APOE phenotype were collected in K_2_EDTA-containing vacutainer tubes (Supplementary Figure S1A). The blood specimens were separated into plasma and stored at −80°C until analysis. The study was conducted in adherence to the Declaration of Helsinki. Written informed consent was obtained from all participants.

### Apolipoprotein measurement for dataset 1

The suitable peptide segments of various Apos we selected meet the criteria for enzymatic cleavage, uniqueness, and lack of known mutations [19,20] (Supplementary Table S1). The 10 μl of the stable isotope-labeled (SIL) internal standard mixture (10 μg/ml) was combined with 5 μl of plasma samples or the calibrators in a 1.5-ml tube containing 75 μl of 1% SDC (w/v) and 50 μl of 100 mmol/l AmBic. This mixture was reduced with 5 μl of 250 mmol/l DTT at 90°C for 60 min at 500 rpm, then mixed with 10 μl of 500 mmol/l IAA for 30 min in the dark at room temperature. Next, 5 μl of 250 mmol/l DTT was added to quench the alkylation reaction, diluting the solution to 1 ml with 0.5% SDC in 100 mmol/l AmBic. Following centrifugation at 10,000×***g*** for 10 min at 4°C, 50 μl of the supernatant was collected and mixed with an equal volume of 0.5% SDC. Subsequently, 1 μg of sequencing-grade Promega trypsin was used for digestion at 37°C on a constant temperature oscillator overnight (18 h). After digestion, 20 μl of 20% formic acid aqueous solution was added to stop the reaction. The mixture was left to stand at room temperature for 5 min before being centrifuged at 13,000×***g*** for 15 min at 4°C.

The LC-QQQ-MS analysis was performed using an AB Sciex Triple Quad 6500+ mass spectrometer equipped with an electrospray ionization source and optimal multiple-reaction monitoring transitions (AB Sciex, Framingham, MA, U.S.A.). An Acquity UPLC HSS T3 column (2.1 × 50 mm, 1.8 μm) was maintained at 45°C at a flow rate of 0.3 ml/min. Mobile phase A consisted of pure water with 0.1% formic acid (FA) and 0.1% dimethyl sulfoxide (DMSO), while mobile phase B was methanol with 0.1% FA and 0.1% DMSO. Data acquisition and analysis were all performed with Analyst 1.7.3 software (AB Sciex) and analyzed with OS 2.1 software (AB Sciex). Detailed information on peptide selection and mass spectrometer parameters for each Apo is provided in Supplemental Table S1.

For method validation, bovine serum albumin (BSA) prepared in phosphate-buffered saline was used as a surrogate matrix to approximate the plasma background. Calibration curves were constructed by analyzing a series of standard peptides at multiple concentration levels in the BSA matrix. For each analyte, the peak area ratio (*Y*) of the peptide to its corresponding SIL internal standard was plotted against the nominal concentration (*X*), and linear regression was performed using a 1/X^2^ weighting factor. The lower limit of quantification was defined as the lowest concentration at which a signal-to-noise ratio of ≥10 was achieved. Method precision and accuracy were evaluated by assessing intra-day and inter-day variability using quality control (QC) samples at three concentration levels (low, medium, and high). QC samples, consisting of both standard solution mixtures and spiked plasma samples, were analyzed in six replicates within a single day for intra-day assessment and over three consecutive days for inter-day evaluation.

### APOE phenotyping confirmed by commercial assay kit for dataset 1

APOE phenotyping in dataset 1 was independently confirmed by Zhongnan Hospital of Wuhan University. Genomic DNA was extracted from whole blood using standard protocols. APOE genotypes were determined by TaqMan allelic discrimination assays targeting SNPs *rs429358 (c.388T>C, Cys130Arg*) and *rs7412 (c.526C>T, Arg176Cys*). These SNPs define six genotypes: ε2/ε2, ε3/ε3, ε4/ε4, ε2/ε3, ε2/ε4, and ε3/ε4. Genotyping was performed using a commercial assay kit (Zhuhai Sinochips Bioscience Co., Ltd.).

### Clinical and laboratory assessment for dataset 1

Routine blood biochemistry tests were conducted following overnight fasting and analyzed by the Department of Laboratory Medicine, Zhongnan Hospital of Wuhan University. Analytes included total cholesterol, triglycerides, high-density lipoprotein (HDL) and low-density lipoprotein (LDL) cholesterol, glucose, kidney function, and liver function in serum were measured by the commercial assay kit on an automatic biochemical analyzer (Roche Cobas 8000 c701, Switzerland).

### CHD participants with selected apolipoproteins from UK Biobank in dataset 2

In dataset 2, we utilized the UKB Olink proteomics database, which provides normalized protein expression (NPX) value of total APOE and selected Apos in 53,026 participants. This research was conducted using the UKB resource under application number 95608. APOE genotypes were identified through whole-exome sequencing using SNPs *rs429358* and *rs7412*. The 31477 individuals with both APOE genotype and Apo expression were included, excluding those with malignant diseases, tumors, and pregnancy. Then ICD10 codes 120–125 were used to identify a total of 3821 participants with ischemic heart disease that were divided into a young (*n* = 148) and aged group (*n* = 3673) based on age threshold (cutoff at 45 years) (Supplementary Figure S1B). The rest of individuals were also divided into a young control group (*n* = 4597) and an elderly control group (*n* = 23,059).

### Statistical analysis

The GraphPad Prism 10.0 software and R environments were used for database management and statistical analysis. The distribution types were evaluated using the Kolmogorov–Smirnov test. Data were expressed as mean ± standard deviation for normally distributed variables and as geometric mean with interquartile range for skewed data. Pearson correlation was applied to assess associations between Apos and clinical biochemical indicators. To minimize confounding factors, propensity score matching (1:2 ratio, caliper width 0.2) was performed for HDL, LDL, and sex between the young and elderly CHD patients. Group differences were analyzed using Mann–Whitney test for nonparametric statistics with false discovery rate corrections. Categorical variables were analyzed using Chi-square or Fisher’s exact test due to small sample size of APOE4 group (*n* < 40). For comparisons across multiple groups, two-way ANOVA with Tukey’s multiple comparison test was used, with *q*-values <0.05 considered significant. Multinomial logistic regression was further performed to adjust for covariates that differed in the baseline characteristics, including sex, BMI, clinical biochemistry parameters, and medication use.

A mediation analysis was conducted to evaluate whether selected Apo mediates the association between age and CHD status using the PROCESS macro for SPSS (Version 4.3). The causal path was examined with age as the independent variables, CHD status as the dependent variable, and Apos as the mediator. Mediation was considered statistically significant if the 95% bootstrap confidence interval (CI) did not include zero. For interpretability, regression coefficients from logistic models were exponentiated and presented as odds ratios with 95% CIs.

## Results

### Method development of apolipoproteins in human plasma

**Figure 1:**
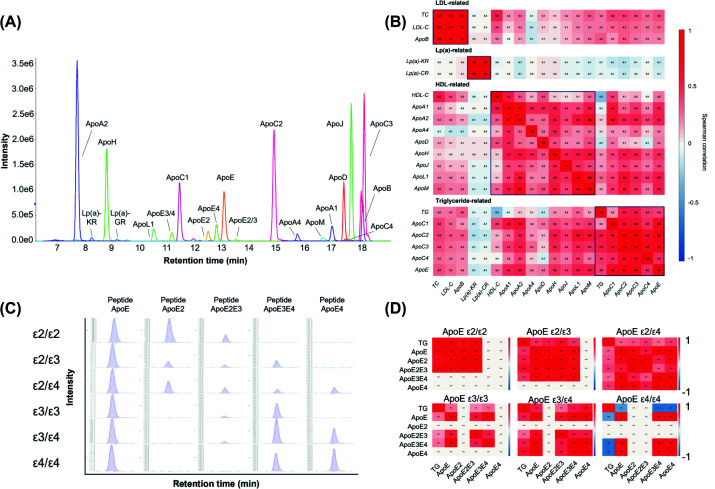
Chromatogram of all measured Apos and their correlation with traditional lipid profiles in CHD patients (**A**) The total ion chromatograms of each peptide were analyzed in a standard solution mixture. (**B**) Correlation between Apos and traditional lipid index in all CHDs. (**C**) The dynamic multiple reaction monitoring chromatograms of each ApoE-specific peptide in six typical APOE genotypes. (**D**) Correlation between ApoE-related specific peptides and traditional lipid index in all CHDs.

The total ApoE, 4 isoform-specific ApoE peptides, and 16 other Apos (e.g., ApoA1, ApoA2, ApoD, ApoM, and Lp(a)) were simultaneously quantified and analyzed by LC-QQQ-MS platform (Figure 1A). The results of method validation are summarized in Table 1. Calibration curves for all quantified peptides exhibited excellent linearity, with coefficients of determination (*R*^2^) exceeding 0.99. The intra-day coefficients of variation (CV) for all quantified and differential peptides ranged from 2.86% to 14.82%, while inter-day CVs ranged from 4.91% to 14.51%. These results demonstrate good repeatability and reproducibility of the analytical methods (Table 1). Furthermore, our reference ranges for most Apos were broadly consistent with those reported in three other studies [21–23], despite differences in population and sample preparation method (Supplementary Table S2).

**Table 1:** Method validation of all the measured Apos

Peptide	*LQC*		*MQC*		*HQC*		Linear	*R* ^2^	Range (ng/ml)^*^
	Intra % CV	Inter % CV	Intra % CV	Inter % CV	Intra % CV	Inter % CV			
ApoA1	5.93%	9.43%	6.94%	8.08%	9.27%	7.59%	*y* = 9.83e^−7^ + 0.0047	0.990	2000–400000
ApoA2	11.59%	7.20%	10.86%	10.82%	11.28%	10.18%	*y* = 0.0027 *x* + 0.20	0.994	1000–200000
ApoA4	8.38%	6.47%	8.51%	8.89%	13.43%	12.62%	*y* = 2.37e^−5^ *x* + 2.76e^−4^	0.992	250–5000
ApoB	5.81%	5.41%	5.23%	7.00%	6.39%	5.91%	*y* = 0.0042 *x* − 0.024	0.998	100-20000
ApoC1	12.81%	11.25%	13.31%	13.72%	12.42%	13.65%	*y* = 2.61e^−4^ *x* + 0.016	0.990	100–20000
ApoC2	4.42%	4.91%	8.70%	10.62%	6.24%	6.29%	*y* = 4.65e^−4^ *x* + 9.56e^−4^	0.999	125–25000
ApoC3	2.86%	4.93%	8.77%	8.65%	4.35%	7.05%	*y* = 1.94e^−4^ *x* + 0.0080	0.999	250–50000
ApoC4	11.99%	13.29%	14.64%	13.22%	10.27%	10.71%	*y* = 3.35e^−5^ *x* + 1.004e^−4^	0.990	250–50000
ApoD	14.82%	13.54%	3.82%	9.96%	14.60%	9.36%	*y* = 4.65e^−4^ *x* + 0.018	0.999	250–50000
ApoE	3.98%	5.43%	5.02%	6.33%	3.38%	6.13%	*y* = 6.17e^−5^ *x* + 1.25e^−4^	0.993	25–5000
ApoH	7.39%	6.77%	8.49%	7.53%	9.66%	9.00%	*y* = 9.13e^−4^ *x* + 0.075	0.991	100–20000
ApoJ	13.20%	10.68%	8.76%	9.59%	8.97%	9.69%	*y* = 1.11e^−4^ *x* + 0.030	0.991	250–50000
ApoL1	12.79%	9.52%	13.09%	13.46%	8.22%	8.16%	*y* = 0.0044 *x* + 0.010	0.994	50–10000
ApoM	11.92%	12.15%	12.11%	14.20%	12.50%	11.74%	*y* = 0.0015 *x* + 0.046	0.987	25–5000
Lp(a)-CR	13.95%	9.03%	9.58%	10.43%	12.69%	14.51%	*y* = 0.0076 *x* + −0.0012	0.993	2–400
Lp(a)-KR	9.52%	12.37%	5.89%	5.34%	9.59%	7.30%	*y* = 0.023*x* + −0.019	0.994	25–5000

Abbreviations: LQC, low concentration of the quality control; MQC, middle concentration of the quality control; HQC, high concentration of the quality control. Lp(a)-KR, apolipoprotein(a) Kringle-IV-type2 repeat peptide; CR, apolipoprotein(a) conserved-region peptide.

* The concentration here refers to the concentration of peptide segment.

Correlation analysis between Apos and clinical lipid index was performed from the cohort (Figure 1B). Plasma total cholesterol and LDL levels are highly correlated with ApoB. The conserved peptide and the variable KIV2 peptide of Lp(a) showed a high degree of correlation with each other. HDL-C was positively correlated with ApoA1/A2/A4/D/H/L1/M/J. TG were significantly associated with ApoC1/C2/C3/C4 and total ApoE.

Based on the presence of isoform-specific peptides, participants were classified into six APOE genotypes (Figure 1C). Among these peptides, the ApoE peptide is common to all APOE isoforms, while the ApoE2 peptide is unique to ε2 genotypes. The ApoE2E3 peptide is shared by ε2 and ε3 genotypes, the ApoE3E4 peptide is shared by both ε3 and ε4 genotypes, and the ApoE4 peptide is unique to ε4 genotypes. Genotype results from LC-QQQ-MS were fully consistent with those from the TaqMan assay in all 293 patients, confirming the accuracy of the LC-QQQ-MS approach for identifying the APOE genotypes (Supplementary Table S3).

Correlation analysis of ApoE peptides and lipid indices demonstrated that, in individuals with the ε2/ε2 genotype, TG was highly positively correlated with total ApoE, ApoE2, and ApoE2E3 peptide (Figure 1D). In the ε3/ε3 genotype—which lacks ApoE2 and ApoE4 peptides—TG was positively correlated with total ApoE, ApoE2E3, and ApoE3E4 peptides. Conversely, in the ε4/ε4 genotype, which lacks ApoE2 and ApoE2E3 peptides, TG showed significant negative correlations with total ApoE, ApoE3E4, and ApoE4 peptides. Taken together, we developed a robust method for simultaneous quantification of APOE isoforms and Apos in 5 μl plasma.

### Distribution of apolipoproteins between young and elderly CHD patients in dataset 1

A total of 293 CHD patients were enrolled from Zhongnan Hospital in this retrospective cohort study (Supplementary Figure S1A). Among them, 75 subjects (male, 84%) were younger than 45 years, and 218 subjects (male, 64.7%) were older than 45 years (Supplementary Table S4). The proportion of males was higher in the younger group, while common comorbidities were more prevalent among older CHD patients. Compared with older patients, younger individuals had higher elevated lipid levels, including TC, TG, and LDL-C. After performing 1:2 propensity score matching (PSM) analysis to adjust for sex, HDL, and LDL levels, no significant differences in lipid profiles were observed between the matched groups, while younger CHD patients exhibited more obvious liver and kidney dysfunction (Table 2). The median ages were 39 years in the young CHD group and 63 years in the older CHD group.

**Table 2:** Baseline characteristics of matched young and aged CHD patients in dataset 1

	All CHD (***n*** = 293)	PSM (1:2)		
		Young CHD (***n*** = 60)	Aged CHD (***n*** = 120)	*P*-value
Sex, man (%)	204 (69.6)	49 (81.6)	83 (69.2)	0.075
Age-years	57 ± 15	37.7 ± 5.6	63.2 ± 10.3	<0.001
Median (IQR)	58 (45-69)	39 (36–42)	63 (55–70)	<0.001
**Disease history (*n*, %)**				
Hypertension	162 (54.5)	18 (30.0)	69 (57.5)	0.002
Dyslipidemia	93 (31.3)	24 (40.0)	35 (29.2)	0.182
Stroke	75 (25.3)	4 (6.6)	32 (26.7)	<0.001
Diabetes	38 (12.8)	3 (5.0)	22 (18.3)	0.008
**Clinical biochemistry**				
TC (mmol/l)	4.5 ± 1.2	4.9 ± 1.1	4.6 ± 1.2	0.178
TG (mmol/l)	1.7 ± 1.1	1.8 ± 0.7	1.7 ± 1.0	0.877
HDL-C (mmol/l)	1.2 ± 0.3	1.2 ± 0.3	1.2 ± 0.3	0.710
LDL-C (mmol/l)	2.9 ± 0.9	3.1 ± 0.9	2.9 ± 0.9	0.413
ALT (U/l)	25.3 ± 29.6	38.3 ± 52.8	20.3 ± 12.1	<0.001
Glucose (mmol/l)	6.1 ± 2.2	5.6 ± 2.2	6.5 ± 2.3	0.002
Creatinine (mmol/l)	78.3 ± 41.3	74.4 ± 16.4	78.5 ± 36.8	0.410
eGFR (ml/min/1.73 m^2^)	94.5 ± 23.3	110.8 ± 17.4	89.8 ± 22.2	<0.001
**APOE Group (*n*, %)**				
APOE2	42 (14.3)	8 (13.3)	11 (9.2)	0.679
APOE3	189 (64.5)	40 (66.7)	76 (63.3)	0.724
APOE4	62 (21.2)	12 (20.0)	33 (27.5)	0.389
Medication				
Statin (%)	50 (16.8)	6 (10.0)	17 (14.2)	0.035

Values for continuous and categorical variables are expressed as median (25th; 75th percentile) and percentage, or mean ± standard deviation, respectively. The Kruskal–Wallis test for continuous variables and the chi-square or Fisher’s exact test for categorical variables were used to determine significant difference between groups.

**Note**: Participants were grouped into three APOE categories due to the small sample sizes of ε2/ε2, ε2/ε4, and ε4/ε4 genotypes: APOE2 (ε2/ε2 and ε2/ε3), APOE3 (ε3/ε3), and APOE4 (ε2/ε4, ε3/ε4, and ε4/ε4).

Abbreviations: IQR, interquartile range; CHD, coronary heart disease; PSM, propensity score matching; HDL, high density lipoprotein; LDL, low density lipoprotein; ALT, alanine Transaminase; eGFR, estimated glomerular filtration rate.

Participants were grouped into three APOE categories due to the small sample sizes of ε2/ε2 and ε4/ε4 genotypes by TaqMan assay [24]: APOE2 group (including ε2/ε2 and ε2/ε3 genotypes), APOE3 group (including ε3/ε3 genotype), and APOE4 group (including ε2/ε4, ε3/ε4 and ε4/ε4 genotypes) [25]. The distribution of the three APOE categories did not differ significantly between the two comparable groups.

The differences in Apos excluding ApoE between young and elderly CHD patients before PSM are presented in Supplementary Table S5. Nearly 10 Apos including Lp(a), showed significant differences between these two comparable groups. After PSM matching (Table 3), five Apos (including ApoA2/C2/J/L1/M) were significantly reduced in the elderly CHD group. We further did multivariable analysis for five selected Apos by adjusting confounding factors that showing significant differences in baseline characteristics (disease history of hypertension, stroke, diabetes, levels of ALT, glucose, eGFR, and medication use). Only ApoL1 still showed statistically significance between the comparable two groups (ApoA2, *P*_adj_ = 0.622; ApoC2, *P*adj = 0.131; ApoJ, *P*_adj_ = 0.123; ApoL1, *P*_adj_ < 0.001; ApoM, *P*_adj_ = 0.252).

**Table 3:** Concentrations of Apos in matched young and aged CHD patients in dataset 1

ConC (ng/ml)	All CHD (*n* = 293)	PSM (1:2)		
		Young CHD (*n* = 60)	Aged CHD (*n* = 120)	*P*-value
ApoA1	39964 ± 12089.2	37985.2 ± 8356.2	40786.4 ± 13218.5	0.571
ApoA2	18958.9 ± 4899.7	20426.5 ± 4421.5	18717.9 ± 4896.9	0.032
ApoA4	1251.5 ± 509.0	1183.5 ± 396.1	1250.5 ± 503.1	0.360
ApoB	1269.4 ± 473.9	1411.6 ± 467.4	1267.3 ± 439	0.053
ApoC1	1713.7 ± 527.2	1742.3 ± 404.3	1714.4 ± 578	0.983
ApoC2	3065.5 ± 1503.0	3690.4 ± 1658.9	3004.2 ± 1361.5	0.014
ApoC3	5864.3 ± 2662.9	6439.4 ± 2482.8	5982.9 ± 2899.6	0.469
ApoC4	33.4 ± 21.7	33.7 ± 16.3	35.3 ± 22.4	0.291
ApoD	1281.7 ± 437.9	1336 ± 432.7	1309.1 ± 481.5	0.842
ApoE	1229.8 ± 603.0	1288 ± 458.6	1161.6 ± 474.1	0.273
ApoH	2324.3 ± 604.8	2451.5 ± 533.2	2334.4 ± 626.9	0.266
ApoJ	3678.7 ± 1009.4	4060 ± 943.4	3588.1 ± 964.3	0.007
ApoL1	221.3 ± 79.6	276 ± 79.5	206.8 ± 66.8	<0.001
ApoM	422.8 ± 155.5	485.6 ± 145.3	418.3 ± 145.1	0.007
Lp(a)-KR	1102.2 ± 1262.4	862.6 ± 919.6	1243.6 ± 1438	0.118
Lp(a)-CR	30.8 ± 42.8	20.2 ± 22.6	35.3 ± 49.3	0.080

Abbreviations: CHD, coronary heart disease; PSM, propensity score matching; Lp(a)-KR, apolipoprotein(a) Kringle-IV-type2 repeat peptide; CR, apolipoprotein(a) conserved-region peptide. ConC: concentration of each peptide.

**Figure 2:**
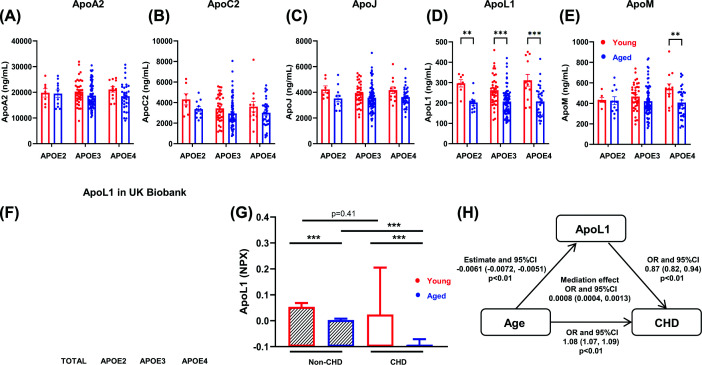
Significant changed Apos in different APOE phenotypes of young and aged CHD patients in two datasets (**A–E**) The concentration of five Apos in young and aged CHD patients across three APOE subtypes. Statistical significance was evaluated by two-way ANOVA with Tukey’s test with multiple comparison correction. (**F**) The violin plot for the ApoL1 distribution in different APOE phenotypes of young and aged CHD patients in UKB after adjusted confounding factors. (**G**) The median and 95% CI of ApoL1 in non-CHD and CHD populations across different ages in UKB after adjusting for confounding factors. (**H**) Mediation of ApoL1 in the association of age and CHD status. OR: odds ratio; 95% CI: 95% confidence interval; *P < 0.05, **P < 0.01, ***P < 0.001.

To further investigate the differences in Apo levels among young and elderly CHD patients with different APOE genotypes, the distribution of the above 5 Apos (ApoA2/C2/J/L1/M) in APOE2, APOE3, and APOE4 groups were compared (Figure 2A–E). After multiple corrections using two-way ANOVA, ApoL1 levels were found to be significantly lower in elderly CHD patients compared with the younger counterparts, regardless of the APOE genotype. Additionally, while ApoM levels were reduced in elderly CHD patients carrying the APOE ε4 allele, the other three Apos showed no significant differences across APOE subtypes.

### Effects of APOE phenotype on apolipoproteins in young and aged CHD patients from the UK Biobank

To further verify the generalizability of our findings, we analyzed a cohort of 3821 CHD patients from the UKB who had available Olink Apo NPX values and APOE genotype data (Supplementary Figure S1B), which allowed us to further evaluate the effect of APOE genotypes on ApoL1 levels in an independent and ethnically diverse cohort.

The baseline characterization of CHD patients in these two groups was presented in Table 4. The proportion of current smokers exceeded 60% in both groups. The elderly CHD patients were more likely to be hypertensive and predominantly of Caucasian ethnicity. The young CHD patients had higher levels of alanine aminotransferase, TC, and LDL-C.

**Table 4:** Baseline characteristics of young and aged CHD patients in UK Biobank

	Young CHD (*n* = 148)	Aged CHD (*n* = 3673)	*P-*value
**Demographic characteristics**			
Age (years)	44 (42, 45)	62 (57, 66)	<0.001
Sex (Male, %)	97 (65.5)	2304 (62.7)	0.488
Ethnic (Caucasian, %)	106 (71.6)	3095 (84.3)	<0.001
Ever smoked (*n*, %)	94 (63.5)	2472 (67.3)	0.336
BMI (Kg/m^2^)	28.7 (25.3, 31.9)	28.2 (25.6, 31.6)	0.750
SBP (mmHg)	132 (122.8, 145.3)	142 (130, 156)	<0.001
**Clinical Biochemistry**			
TC (mmol/l)	5.4 (4.7, 6.3)	5.2 (4.3, 6.2)	0.073
TG (mmol/l)	1.8 (1.1, 2.5)	1.7 (1.2, 2.4)	0.674
HDL (mmol/l)	1.2 (1, 1.3)	1.2 (1, 1.5)	0.039
LDL (mmol/l)	3.5 (2.8, 4)	3.2 (2.5, 3.9)	0.013
Lipoprotein(a) (mmol/l)	25.2 (9.4, 80.5)	24.2 (10.1, 73.1)	0.765
ALT (U/L)	23.1 (17, 33.5)	22 (16.8, 30.2)	0.034
Glucose (mmol/l)	4.8 (4.5, 5.2)	5 (4.7, 5.5)	0.119
Creatinine (µmol/l)	71.6 (63.5, 84.9)	75.2 (65, 86.5)	0.819
**ApoE group**			
APOE2 (*n*, %)	12 (8.1)	399 (10.9)	0.344
APOE3 (*n*, %)	98 (66.2)	2126 (57.9)	0.051
APOE4 (*n*, %)	38 (25.7)	1148 (31.3)	0.174
**Medication**			
Anti-cholesterol (*n*, %)	26 (17.6)	1292 (35.2)	<0.001
Anti-blood pressure (*n*, %)	25 (16.9)	1186 (32.3)	<0.001
Anti-diabetes (*n*, %)	3 (2)	87 (2.4)	0.999
**Olink data**			*P*_adj_ value^*^
ApoL1 (NPX)	0.02 (−0.25, 0.49)	−0.09 (−0.38, 0.21)	<0.001

Values for continuous and categorical variables are expressed as median (25th, 75th percentile) and percentage, respectively. The Kruskal–Wallis test for continuous variables and the chi-square test for categorical variables were used to determine significant difference between groups.

**Note**: Participants were grouped into three APOE categories due to the small sample sizes of ε2/ε2, ε2/ε4, and ε4/ε4 genotypes: APOE2 (ε2/ε2 and ε2/ε3), APOE3 (ε3/ε3), and APOE4 (ε2/ε4, ε3/ε4, and ε4/ε4).

Abbreviations: CHD, coronary heart disease; BMI, body mass index; HDL, high-density lipoprotein; LDL, low-density lipoprotein; ALT, alanine aminotransferase; ApoL1, apolipoprotein L1, NPX, normalized protein expression; SBP, systolic blood pressure; TC, total cholesterol; TG, triglycerides.

* Multinomial logistic regression was further performed to adjust for all covariates presented in the tables, including sex, BMI, clinical biochemistry parameters, and medication.

Consistent with our findings from dataset 1, plasma APOL1 levels were significantly reduced in all European aged CHD patients. Upon stratification by APOE genotype, the APOL1 levels were notably decreased in individuals with ε3 and ε4 genotypes, whereas its impact appears insignificant in those with the ε2 allele (Figure 2F).

To further determine whether the differences in APOL1 levels between young and elderly CHD patients were due to the disease itself or to aging, we included non-CHD participants from the UKB as control groups (Supplementary Table S6). The results showed that APOL1 levels were significantly decreased in elderly CHD patients compared with their age-matched controls, whereas no significant changes were observed in young CHD patients (Figure 2G).

Following the mediation analysis, ApoL1 was found to statistically mediate the association between age and CHD status. Age was negatively associated with ApoL1, and higher ApoL1was linked to lower odds of CHD status. Age remained positively associated with CHD after accounting for ApoL1. Bootstrap analysis confirmed a significant indirect effect of age on CHD status via ApoL1, supporting its partial mediation effects. These patterns were consistent in both crude and adjusted models by adjusting confounding factors including lipid profiles (HDL, LDL, TC, and TG), creatinine, alanine transaminase, glucose and body mass index (Figure 2H and Supplementary Figure S2). Taken together, our findings suggest that the decreased levels of APOL1 in CHD patients, particularly those carrying the APOE ε3 or ε4 allele.

## Discussion

In the present study, we developed a chromatography method capable of simultaneously detecting Lp(a), APOE genotype-specific peptides, and 14 other Apos. We found that an LC-QQQ-MS-based platform is not only effective in distinguishing different APOE isoforms qualitatively but also provides quantitative insights into other Apos in young and elderly CHD patients in relation to different APOE subtypes. Furthermore, plasma levels of ApoL1 were notably reduced in elderly CHD patients, particularly among those with the APOE ε3 and ε4 genotypes.

APOE phenotyping is known to play an important role in CHD risk assessment [26]; however, traditional genotyping methods are primarily qualitative and do not distinguish between the expression levels of different APOE isoforms. Furthermore, total APOE protein quantification alone fails to obtain isoform-specific changes. To address this limitation, we employed a targeted LC-QQQ-MS approach that enables both qualitative genotyping and quantitative peptide-level analysis of APOE isoforms. The reference ranges in our works for most Apos were broadly consistent with other previous studies [21–23]. Furthermore, the APOE classification obtained from the mass spectrometry platform in our cohort was fully consistent with those determined using a commercial genotyping assay, supporting the reliability of our method. While quantitative peptide-level analysis of APOE isoforms has been mainly reported in neurodegenerative diseases, with limited application in cardiovascular diseases. For instance, Deza-Lougovski et al. reported that, among 12,532 adults, higher circulating ApoE4 levels were associated with better cognitive performance in Alzheimer’s disease patients [27]. Similarly, Edlund found plasma ApoE3 levels negatively correlated with blood glucose levels in Alzheimer’s disease patients [28]. Baker-Nigh observed that cerebrospinal fluid APOE isoform levels rather than plasma ApoE levels showed a stronger correlation with amyloid-beta pathology in Alzheimer’s disease [29]. In contrast with the neurological focus of prior studies, our work reveals a cardiovascular-specific pattern with ApoE-specific peptides. We found that ApoE4-specific peptides were significantly decreased in elderly CHD patients with ApoE4 subtype, even when total ApoE levels remained unchanged (Supplementary Figure S3). These findings suggest that isoform-specific quantification of ApoE peptides may provide additional insights than total ApoE levels.

APOE genetic polymorphisms are a major determinant of plasma lipid profiles and, consequently, cardiovascular disease risk. This link is robustly supported by comprehensive meta-analytic data, which indicate that carriage of the APOE ε4 allele elevates the risk of CHD by 46%. In contrast, the ε2 allele does not confer a significant overall protective effect against CHD [30]. Intriguingly, this ε2-associated protection appears ethnically restricted, being observed in Caucasian populations but not Mongolian population [30], suggesting population-specific interactions modulate its phenotypic expression. This disparity may stem from isoform-specific interaction between APOE and its receptors like low-density lipoprotein receptor, whose binding affinity is markedly weaker for APOE2 than for APOE3 and APOE4 [31,32]. Furthermore, *in vivo* kinetic studies of ^2^H^3^-leucine enrichment have revealed distinct metabolic fates for each isoform: APOE2 exhibits a slower catabolic rate, whereas APOE4 has a reduced production rate compared with APOE3 [33]. Collectively, these kinetic characteristics may help explain our observations: the decreased total ApoE and ApoE2/E3 peptide levels in ε4 carriers, alongside increased ApoE3/E4 peptide levels in ε4 homozygotes, likely reflects the consequences of fundamental isoform-specific properties on protein metabolism and clearance.

Apart from ApoE, the levels of ApoL1 exhibited the most significant change among all Apos in both groups of CHD patients. ApoL1 is an important mediator of HDL components and plays a key role in lipid transport and metabolism. ApoL1 genotype risk variants are known to contribute to the etiology of kidney disease [34]. However, the association between ApoL1 risk variants and cardiovascular disease remains unclear or even controversial in previous studies. For example, the Jackson Heart Study [35,36] and the Women’s Health Initiative Study [37] reported that ApoL1 risk variants were associated with an increased risk of cardiovascular events. On the other hand, the Systolic Blood Pressure Intervention Trial [38] and Atherosclerosis Risk in Communities [39] studies found no significant association between APOL1 gene variants and blood pressure or the incidence of atherosclerotic disease. Recently, proteomics-driven studies have revealed that plasma levels of ApoL1 were positively associated with bone mineral density in an osteoporosis prospective study [40]. Moreover, decreased plasma ApoL1 levels have been regarded as one of the indicators for identifying children with hypertrophic cardiomyopathy at high risk of sudden cardiac death [41]. Additionally, ApoL1 has been found to be negatively associated with age in a longitudinal healthy aging cohort, though this aging-related protein showed no association with hypertension, type 2 diabetes mellitus, hepatitis, or renal disease [42]. In our study, we found that plasma ApoL1 levels were significantly reduced in elderly individuals with CHD, irrespective of ApoE genotype. Despite observations linking APOL1 polymorphisms to human plasma ApoE levels [43], the underlying relationship requires more robust evidence for further confirmation.

In our study, we found that plasma APOL1 levels were significantly reduced in elderly individuals with CHD. Given that previous studies have shown a strong association between APOL1 and age [42], our mediation analysis revealed that ApoL1 was statistically associated with the relationship between age and CHD status even after adjusting for a series of confounding factors (Figure 2H and Supplementary Figure S2). Although the regulation of APOE genotype on the expression of APOL1 has not been extensively reported in previous studies, plasma APOE levels have been found to be influenced by the polymorphic genotypes of the APOL1 gene [43], implying a bidirectional regulation between APOE and APOL1 that needs to be further investigated in future works.

Last but not least, although it’s generally believed that the CHD patients under the age of 45 are considered early-onset CHD [1]. However, the age definition for young and elderly CHD varies across different studies, ranging from 35 to 55 years. Some studies suggest that the definition of young CHD should be based on gender stratification (male <45 years and female <55 years) [44]. While others define young CHD as occurring in individuals under 50 years of age [45]. In our current discovery cohort, we set the cutoff at 45, as the sample size of elderly CHD patients older than 55 was relatively small after 1:2 PSM matching. We also tested different cutoff age values and found the distribution of APOL1 between premature and elderly CHD patients remained consistent across different age criteria in the validated UKB dataset (Supplementary Figure S4). This cut-off sensitivity analysis highlighted the decreased APOL1 in older CHD patients, especially among those carrying the APOE ε3 and ε4 genotypes.

However, the present study also has several limitations. First, the plasma Apo data were derived exclusively from Chinese populations, while the UKB-PPP plasma proteomic data are based on a British cohort, which includes only a small proportion of Chinese individuals (approximately 0.3%). This potential etiological heterogeneity between Eastern and Western populations may have led to the omission of some key differentially expressed protein. Second, the Olink data from the UKB cohort only provide normalized values for total APOE without information on specific APOE isoform peptides, making it hard to validate the application of isoform-specific APOE peptide quantification in an independent population. Third, the number of CHD cases included is relatively small even if our results get validation from an external cohort in UKB. Further validation through large-scale, multicenter prospective studies is warranted in the future.

## Conclusion

Our results suggested that APOE polymorphisms affect the plasma Apo profiles and that ApoL1 is associated with older CHD patients carrying the APOE E3 and E4 genotypes.

## Supplementary Material

Supplementary Figures S1-S4 and Tables S1-S6

## Data Availability

The genotype data was upload to data repository platform Zenodo (https://zenodo.org/records/17367186) [24].

## Abbreviations

Apo: apolipoprotein
ApoE: apolipoprotein E
BSA: bovine serum albumin
CHD: coronary heart disease
CI: confidence interval
CV: coefficients of variation
DMSO: dimethyl sulfoxide
FA: formic acid
HDL: high-density lipoprotein
LC-QQQ-MS: liquid chromatography triple quadrupole mass spectrometers
LDL: low-density lipoprotein
Lp(a): lipoprotein(a)
NPX: normalized protein expression
PSM: propensity score matching
QC: quality control
SIL: stable isotope-labeled
UKB: UK Biobank
