# Supplementary material for: Apolipoprotein variations across APOE genotypes in young and elderly patients with coronary heart disease
Source: Biosci Rep. 2026 Jun 12;46(6):BSR20260258. doi: 10.1042/BSR20260258 (PMC13270160; doi:10.1042/BSR20260258)
Supplement: Supplementary Figures S1-S4 and Tables S1-S6 [file BSR-2026-0258_supp.pdf]

Supplemental Information

Apolipoprotein variations across APOE genotypes in young and elderly patients with coronary heart disease

Zhang et al

Supplementary Figures and Tables

Supplementary Figure S1. Flowchart of two datasets from cohort study and UK Biobank**.**

Supplementary Figure S2. Mediation of ApoL1 in the associations of age and CHD before adjusting the confounding factors in UK Biobank.

Supplementary Figure S3. Distribution of ApoE specific peptide in different APOE genotypes between young and aged CHD patients in dataset 1.

Supplementary Figure S4. Distribution of APOL1 of total and three APOE subtypes using different age cutoff for young CHD patients in UK Biobank. (A) Threshold age for young CHD: male ≤ 45 years, female ≤ 55 years; (B) Threshold age for young CHD: male/female ≤ 50 years.

Supplementary Tables

Supplementary Table S1. The MS parameter of each peptide and its internal standards.

Supplementary Table S2. Comparison of apolipoprotein concentrations measured in this study with reported reference values.

Supplementary Table S3. APOE subtype by Taqman assay and LC-QQQ-MS measurement of ApoE peptides in all patients.

Supplementary Table S4. Baseline characteristics of all young and aged CHD patients in dataset 1.

Supplementary Table S5. Concentration of apolipoproteins in all young and aged CHD patients in dataset 1.

Supplementary Table S6. Demographic and clinical characteristics of the CHD subjects from UK Biobank according to age.

Supplementary Figure S1. Flowchart of two datasets from cohort study and UK Biobank**.**

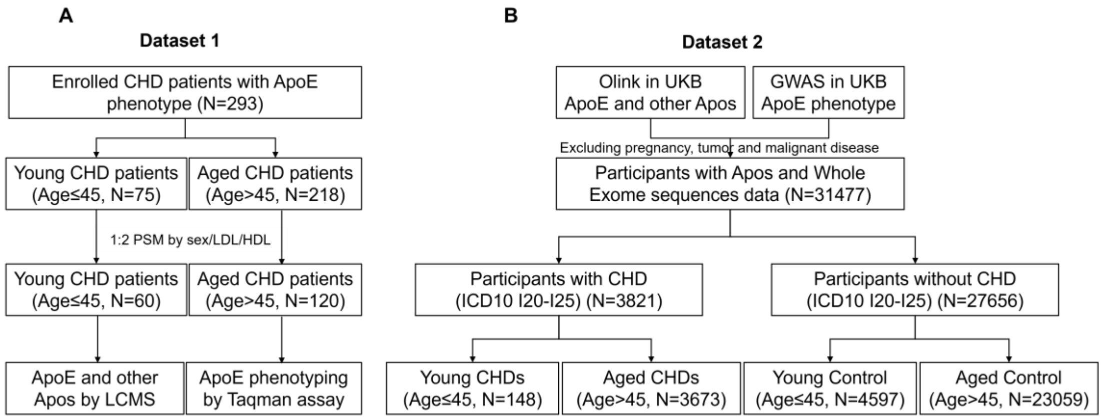

Supplementary Figure S2. Mediation of ApoL1 in the associations of age and CHD before adjusting the confounding factors in UK Biobank.

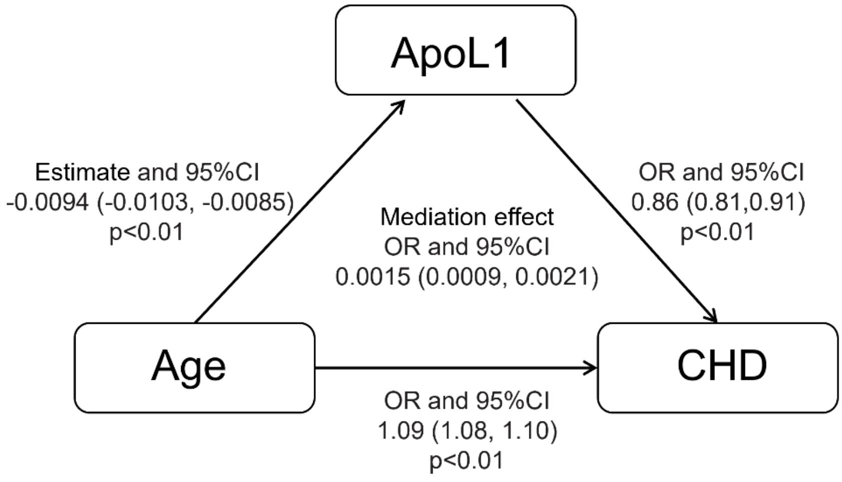

Supplementary Figure S3. Distribution of ApoE specific peptide in different APOE genotypes between young and aged CHD patients in dataset 1.

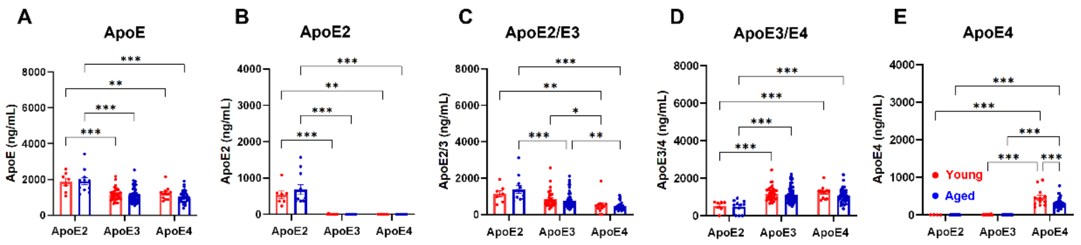

(A-E) Distribution of ApoE specific peptide in different APOE genotypes between young and aged CHD patients. * P<0.05, ** P<0.01, ***P<0.001.

Supplementary Figure S4. Distribution of APOL1 of total and three APOE subtypes using different age cutoff for young CHD patients in UK Biobank.

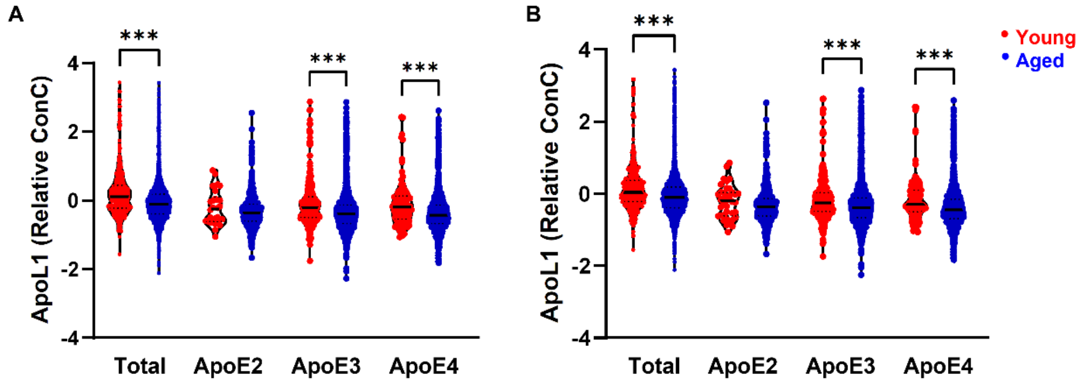

(A) Threshold age for young CHD: male ≤ 45 years, female ≤ 55 years; (B) Threshold age for young CHD: male/female ≤ 50 years. *** p < 0.001.

Supplementary Table S1: The MS parameter of each peptide and its internal standards.

Peptide	Peptide name	Protein MW （Kda）	Peptide MW	Q1	Q3	DP	CE	SIL
VQPYLDDFQK	ApoA1	30.8	1252.4	627.1	1025.7	44	23	ApoA1-IS
					1008.4	23	24	
VQPYLDDFQK*	ApoA1-IS	/	1260.4	631.1	1033.7	46	21.3	/
					1016.4	40	25.6	
SPELQAEAK	ApoA2	11.2	972.1	487.1	442.8	87.3	24	ApoA2-IS
					659.4	44	26	
SPELQAEAK*	ApoA2-IS	/	980.1	491.1	450.8	87.3	24	/
					667.4	44	26	
LTPYADEFK	ApoA4	41.2	1083.2	542.3	772.4	38	24	ApoA4-IS
					435.2	40	29.6	
LTPYADEFK*	ApoA4-IS	/	1091.2	550.3	683.6	40	30	/
					925.4	45	28	
FPEVDVLTK	ApoB	515.6	1047.2	524.5	450.5	149.7	23	ApoB-IS
					803.6	139.4	30	
FPEVDVLTK*	ApoB-IS	/	1055.2	528.5	811.6	139.4	30	/
					908.6	60	30	
EFGNTLEDK	ApoC1	9.3	1052.1	527.1	391.3	41	22	ApoC1-IS
					776.4	31	24	
EFGNTLEDK*	ApoC1-IS	/	1060.1	531.1	399.3	41	24	/
					784.4	44	22	
TYLPAVDEK	ApoC2	11.3	1035.1	518.3	658.3	60	24.2	ApoC2-IS
					771.6	68	20.8	
TYLPAVDEK*	ApoC2-IS	/	1043.2	522.3	666.3	128	25	/
					779.6	86	23	
GWVTDGFSSLK	ApoC3	10.9	1195.8	598.9	854.6	60	27.1	ApoC3-IS
					343.2	40	23.6	
GWVTDGFSSLK*	ApoC3-IS	/	/	598.9	854.6	60	27.1	/
					343.2	40	23.6	
ELLETVVNR	Apoc4	43.1	1071.6	536.8	588.3	70	30	ApoC1-IS
					388.2	70	30	
NILTSNNIDVK	ApoD	21.3	1230	616	890.6	131	32	ApoD-IS
					789.4	146	31	
NILTSNNIDVK*	ApoD-IS	/	/	620	898.6	144	28	/
					797.4	102	35	
LGPLVEQGR	ApoE	36.2	967.6	484.8	588.5	60	30.6	ApoE-IS
					701.6	50	31.1	
LGPLVEQGR*	ApoE-IS	/	/	489.8	598.5	60	30.8	/
					711.6	50	31.7	
C[+57]LAVYQAGAR	ApoE2	/	/	554.78	835.4	70	28	ApoE-IS
					764.4	70	28	

LGADMEDVC[+57] GR	ApoE2/E3	/	/	611.8	981.4<br>866.4	70<br>70	28<br>28	ApoE-IS
LGADMEDVR	ApoE4	/	/	503.5	274.2<br>892.7	50<br>50	36<br>20	ApoE-IS
LAVYQAGAR	ApoE3/E4	/	/	475	502.3<br>665.4	46<br>46	26<br>24	ApoE-IS
ATVVYQGER	ApoH	38.3	1022	512	652.4<br>751.4	66<br>75	22<br>15	ApoH-IS
ATVVYQGER*	ApoH-IS	/	/	517	662.4<br>761.4	52<br>67	33<br>15	/
VTTVASHTSDSDV PSGVTEVVVK	ApoJ	51.1	1542.8	772.4	507.9	50	30.2	ApoE-IS
ALDNLAR	ApoL1	42.0	771.4	386.9	473.4<br>588.5	53<br>93	27<br>17	ApoA1-IS
FLLYNR	ApoM	21.2	825.0	413.6	565.3<br>452.3<br>669.4	125.1<br>100<br>70	125.1<br>100<br>30	ApoA1-IS
GISSTTVTGR	Lp(a)-CR	/	978.1	489.9	808.3<br>533.3	30<br>40	21.8<br>25.2	Lp(a)-IS
GISSTTVTGR*	Lp(a)-CR-IS	/	988.1	495.3	543.2<br>818.2	30<br>31	22.5<br>22.8	/
GTYSTTVTGR	Lp(a)-KR	/	1042.1	521.8	721.5<br>634.5	58.7<br>57.1	27<br>26	Lpa-kiv2-IS
GTYSTTVTGR*	Lp(a)-KR-IS		1052.1	526.8	644.5<br>895.5	58.7<br>57.1	27<br>26	/

* Are the internal standards peptides; [C+57] indicates carbamydomethyl-cysteine (+57)

Keys: Lp(a)-KR, apolipoprotein(a) Kringle-IV-type2 repeat peptide; CR, apolipoprotein(a) conserved-region peptide.

Supplementary Table S2. Comparison of apolipoprotein concentrations measured in this study with reported reference values

Peptide	Peptide MW	Peptide mass ConC (ng/ml)	Protein molar ConC (µmol/L)	RR[1] (µmol/L)	RR[2] (µmol/L)	RR[3] (µmol/L)	RR[4] (µmol/L)
ApoA1	1252.4	39964.0 ± 12089.2	31.9 ± 9.6	50.1-66.0	20.3–82.3	32.9–61.5	25.0–39.2
ApoA2	972.1	18958.9 ± 4899.7	19.5 ± 5.0	41.3-53.9	18.4–87.6	29–49.7	12.5–21.5
ApoA4	1083.2	1251.5 ± 509.0	1.1 ± 0.4	1.9-3.2	0.5–4.2	0.4–1.7	ND
ApoB	1047.2	1269.4 ± 473.9	1.2 ± 0.4	1.5-2.4	0.3–4.4	0.8–3.2	0.7–1.7
ApoC1	1052.1	1713.7 ± 527.2	1.6 ± 0.5	7.2-10.2	1.9–18.6	2.2–10.4	2–4.6
ApoC2	1035.1	3065.5 ± 1503.0	2.9 ± 1.4	ND	ND	1.2–7.2	0.4–5.6
ApoC3	1195.8	5864.3 ± 2662.9	4.9 ± 2.2	16.1-22.5	0.9–41.1	1.4–10.5	0.8–8.8
ApoC4	1071.6	33.4 ± 21.7	0.03 ± 0.02	ND	ND	ND	ND
ApoD	1230	1281.7 ± 437.9	1.0 ± 0.3	ND	ND	ND	0.5–1.1
ApoE	967.6	1229.8 ± 603.0	1.2 ± 0.6	1.1-1.8	0.3–2.7	0.4–1.6	0.1–2.9
ApoH	1022.0	2324.3 ± 604.8	2.2 ± 0.5	ND	ND	ND	1.8–2.8
ApoJ	1542.8	3678.7 ± 1009.4	2.3 ± 0.6	4.2-5.6	ND	ND	3–5
ApoL1	771.4	221.3 ± 79.6	0.2 ± 0.1	ND	ND	ND	0.5–0.9
ApoM	825.0	422.8 ± 155.5	0.5 ± 0.1	ND	ND	ND	0.4–0.6
Lp(a)-CR	978.0	30.8 ± 42.8	0.03 ± 0.04	ND	ND	ND	0.01–0.13

NOTE: Apolipoprotein concentrations measured in this study were quantified by LC–QQQ-MS using surrogate peptides. Protein molar ConC (µmol/L) were derived from peptide mass concentrations by division with the corresponding peptide molecular weight (MW), to enable comparison with literature.

Keys: #RR, reference range. *ND, not detected. ConC, Concentration. CR, apolipoprotein(a) conserved-region peptide.

Supplementary Table S3. APOE subtype by Taqman and LC-QQQ-MS measurement of ApoE peptides in all patients.

Group	APOE subtype by Taqman	ApoE (ng/ml)	ApoE2 (ng/ml)	ApoE2/E3 (ng/ml)	ApoE3/E4 (ng/ml)	ApoE4 (ng/ml)	APOE subtype by LC-MS/MS
Young CHD	ε2/ε2	5158.1	1723.7	2590.4	NA	NA	ε2/ε2
Young CHD	ε2/ε2	3899.1	1596.7	2475.0	NA	NA	ε2/ε2
Young CHD	ε2/ε2	2576.2	1086.2	1940.4	NA	NA	ε2/ε2
Young CHD	ε2/ε3	2012.8	578.3	1424.4	674.7	NA	ε2/ε3
Young CHD	ε2/ε3	2029.1	408.1	967.3	976.2	NA	ε2/ε3
Young CHD	ε2/ε3	1661.5	353.6	983.5	712.4	NA	ε2/ε3
Young CHD	ε2/ε3	2330.3	588.7	1135.9	713.3	NA	ε2/ε3
Young CHD	ε2/ε3	1081.8	232.6	542.8	381.3	NA	ε2/ε3
Young CHD	ε2/ε3	1273.6	366.3	697.8	442.7	NA	ε2/ε3
Young CHD	ε2/ε3	2135.1	562.2	1281.6	724.2	NA	ε2/ε3
Young CHD	ε3/ε3	1679.7	N/A	918.5	1843.3	NA	ε3/ε3
Young CHD	ε3/ε3	2169.6	N/A	1257.1	2442.9	NA	ε3/ε3
Young CHD	ε3/ε3	1227.5	N/A	1329.1	1272.7	NA	ε3/ε3
Young CHD	ε3/ε3	1132.1	N/A	1849.0	1116.7	NA	ε3/ε3
Young CHD	ε3/ε3	559.9	N/A	856.3	555.0	NA	ε3/ε3
Young CHD	ε3/ε3	1201.8	N/A	733.6	1329.1	NA	ε3/ε3
Young CHD	ε3/ε3	896.3	N/A	538.1	1017.6	NA	ε3/ε3
Young CHD	ε3/ε3	1330.2	N/A	712.4	1336.1	NA	ε3/ε3
Young CHD	ε3/ε3	750.7	N/A	410.2	692.5	NA	ε3/ε3
Young CHD	ε3/ε3	1202.3	N/A	544.8	1146.8	NA	ε3/ε3
Young CHD	ε3/ε3	2386.9	N/A	1146.1	2611.4	NA	ε3/ε3
Young CHD	ε3/ε3	1081.3	N/A	497.4	1132.6	NA	ε3/ε3
Young CHD	ε3/ε3	1282.6	N/A	665.9	1381.1	NA	ε3/ε3
Young CHD	ε3/ε3	900.8	N/A	1518.5	875.7	NA	ε3/ε3
Young CHD	ε3/ε3	911.6	N/A	1029.3	879.6	NA	ε3/ε3
Young CHD	ε3/ε3	973.4	N/A	782.7	933.8	NA	ε3/ε3
Young CHD	ε3/ε3	1038.4	N/A	664.0	908.1	NA	ε3/ε3
Young CHD	ε3/ε3	1204.6	N/A	2579.8	1181.4	NA	ε3/ε3
Young CHD	ε3/ε3	881.4	N/A	1113.8	823.9	NA	ε3/ε3
Young CHD	ε3/ε3	1446.9	N/A	790.7	1592.6	NA	ε3/ε3
Young CHD	ε3/ε3	1263.7	N/A	1078.9	1250.8	NA	ε3/ε3
Young CHD	ε3/ε3	966.4	N/A	531.3	969.9	NA	ε3/ε3
Young CHD	ε3/ε3	678.1	N/A	386.8	740.6	NA	ε3/ε3
Young CHD	ε3/ε3	828.7	N/A	493.2	951.8	NA	ε3/ε3
Young CHD	ε3/ε3	1044.6	N/A	561.9	1033.7	NA	ε3/ε3
Young CHD	ε3/ε3	1176.4	N/A	1138.2	1310.8	NA	ε3/ε3
Young CHD	ε3/ε3	648.3	N/A	1131.3	660.9	NA	ε3/ε3
Young CHD	ε3/ε3	922.5	N/A	517.7	852.7	NA	ε3/ε3

Young CHD	ε3/ε3	1246.7	N/A	755.3	1203.1	NA	ε3/ε3
Young CHD	ε3/ε3	1427.4	N/A	605.1	1370.5	NA	ε3/ε3
Young CHD	ε3/ε3	1296.8	N/A	774.6	1313.0	NA	ε3/ε3
Young CHD	ε3/ε3	1998.1	N/A	1085.0	1808.1	NA	ε3/ε3
Young CHD	ε3/ε3	1664.3	N/A	738.9	1799.2	NA	ε3/ε3
Young CHD	ε3/ε3	1115.3	N/A	885.0	1025.4	NA	ε3/ε3
Young CHD	ε3/ε3	1419.8	N/A	775.8	1446.5	NA	ε3/ε3
Young CHD	ε3/ε3	1270.6	N/A	1034.3	1345.1	NA	ε3/ε3
Young CHD	ε3/ε3	1170.6	N/A	503.5	1207.6	NA	ε3/ε3
Young CHD	ε3/ε3	581.7	N/A	432.0	561.2	NA	ε3/ε3
Young CHD	ε3/ε3	1148.0	N/A	735.8	1032.0	NA	ε3/ε3
Young CHD	ε3/ε3	1415.9	N/A	854.0	1444.9	NA	ε3/ε3
Young CHD	ε3/ε3	1017.1	N/A	1088.1	944.6	NA	ε3/ε3
Young CHD	ε3/ε3	907.6	N/A	612.4	995.9	NA	ε3/ε3
Young CHD	ε3/ε3	684.5	N/A	319.5	677.6	NA	ε3/ε3
Young CHD	ε3/ε3	1571.2	N/A	832.3	1513.9	NA	ε3/ε3
Young CHD	ε3/ε3	1750.4	N/A	931.9	1675.2	NA	ε3/ε3
Young CHD	ε3/ε3	1490.5	N/A	623.5	1372.4	NA	ε3/ε3
Young CHD	ε3/ε3	918.3	N/A	618.5	897.3	NA	ε3/ε3
Young CHD	ε3/ε3	739.6	N/A	410.6	811.9	NA	ε3/ε3
Young CHD	ε3/ε4	1452.0	N/A	497.4	1457.4	454.8	ε3/ε4
Young CHD	ε3/ε4	793.0	N/A	232.4	894.5	212.0	ε3/ε4
Young CHD	ε3/ε4	1426.9	N/A	534.8	1389.4	459.1	ε3/ε4
Young CHD	ε3/ε4	1188.3	N/A	579.1	1286.1	353.3	ε3/ε4
Young CHD	ε3/ε4	936.4	N/A	341.5	905.7	249.8	ε3/ε4
Young CHD	ε3/ε4	2150.2	N/A	1853.0	2031.2	599.6	ε3/ε4
Young CHD	ε3/ε4	1287.8	N/A	601.1	1314.9	491.6	ε3/ε4
Young CHD	ε3/ε4	968.1	N/A	340.3	941.4	297.8	ε3/ε4
Young CHD	ε3/ε4	921.0	N/A	419.5	947.3	353.3	ε3/ε4
Young CHD	ε3/ε4	1376.1	N/A	550.5	1340.8	444.3	ε3/ε4
Young CHD	ε3/ε4	880.9	N/A	358.4	881.2	256.8	ε3/ε4
Young CHD	ε3/ε4	1365.6	N/A	516.3	1319.8	359.2	ε3/ε4
Young CHD	ε3/ε4	874.1	N/A	467.0	819.7	254.6	ε3/ε4
Young CHD	ε3/ε4	1708.4	N/A	468.1	1807.3	510.2	ε3/ε4
Young CHD	ε3/ε4	1215.1	N/A	539.7	1290.2	348.4	ε3/ε4
Young CHD	ε4/ε4	1276.6	N/A	N/A	1308.8	929.4	ε4/ε4
Young CHD	ε4/ε4	1192.0	N/A	N/A	1318.0	877.7	ε4/ε4
Aged CHD	ε2/ε2	2557.3	1314.3	2165.9	N/A	N/A	ε2/ε2
Aged CHD	ε2/ε2	3421.5	1571.7	3114.2	N/A	N/A	ε2/ε2
Aged CHD	ε2/ε2	2370.2	1086.0	1402.5	N/A	N/A	ε2/ε2
Aged CHD	ε2/ε2	1965.8	1120.4	1481.0	N/A	N/A	ε2/ε2
Aged CHD	ε2/ε3	1417.9	445.1	1331.5	659.5	N/A	ε2/ε3
Aged CHD	ε2/ε3	1543.8	374.5	956.2	579.1	N/A	ε2/ε3
Aged CHD	ε2/ε3	4227.3	1361.5	3577.0	1699.0	N/A	ε2/ε3

Aged CHD	ε2/ε3	1350.3	333.1	754.0	474.8	N/A	ε2/ε3
Aged CHD	ε2/ε3	1946.6	705.9	1709.5	771.4	N/A	ε2/ε3
Aged CHD	ε2/ε3	1179.1	357.4	758.0	410.7	N/A	ε2/ε3
Aged CHD	ε2/ε3	1007.0	328.5	736.9	444.5	N/A	ε2/ε3
Aged CHD	ε2/ε3	1524.2	368.1	849.2	606.8	N/A	ε2/ε3
Aged CHD	ε2/ε3	2122.0	444.3	947.3	939.3	N/A	ε2/ε3
Aged CHD	ε2/ε3	1277.6	422.4	1040.9	567.4	N/A	ε2/ε3
Aged CHD	ε2/ε3	792.1	359.0	940.9	290.9	N/A	ε2/ε3
Aged CHD	ε2/ε3	1930.3	346.2	866.2	810.6	N/A	ε2/ε3
Aged CHD	ε2/ε3	1024.2	266.2	688.2	436.3	N/A	ε2/ε3
Aged CHD	ε2/ε3	874.4	286.8	478.1	157.6	N/A	ε2/ε3
Aged CHD	ε2/ε3	1463.6	299.6	763.8	564.2	N/A	ε2/ε3
Aged CHD	ε2/ε4	2494.0	594.9	886.6	857.3	680.6	ε2/ε4
Aged CHD	ε2/ε4	1675.0	421.5	576.0	489.2	341.6	ε2/ε4
Aged CHD	ε2/ε4	1123.7	801.0	1572.5	264.8	215.4	ε2/ε4
Aged CHD	ε2/ε4	2066.6	680.5	888.6	638.4	483.2	ε2/ε4
Aged CHD	ε2/ε4	2885.7	958.0	1471.3	994.8	711.3	ε2/ε4
Aged CHD	ε2/ε4	3170.2	910.2	1083.2	1196.4	801.5	ε2/ε4
Aged CHD	ε2/ε4	3776.7	1023.8	1306.5	1079.6	818.3	ε2/ε4
Aged CHD	ε2/ε4	1664.1	482.5	781.1	508.4	393.7	ε2/ε4
Aged CHD	ε2/ε4	1560.7	731.1	1045.3	445.4	372.7	ε2/ε4
Aged CHD	ε2/ε4	1131.6	319.9	474.4	357.6	241.9	ε2/ε4
Aged CHD	ε2/ε4	2420.4	918.9	1186.1	852.7	644.9	ε2/ε4
Aged CHD	ε2/ε4	1096.1	519.1	714.7	187.7	148.2	ε2/ε4
Aged CHD	ε2/ε4	971.7	74.1	362.5	803.1	182.6	ε2/ε4
Aged CHD	ε3/ε3	783.1	NA	373.1	816.1	NA	ε3/ε3
Aged CHD	ε3/ε3	1491.5	NA	926.0	1396.1	NA	ε3/ε3
Aged CHD	ε3/ε3	1187.5	NA	614.0	1153.6	NA	ε3/ε3
Aged CHD	ε3/ε3	926.2	NA	530.6	893.5	NA	ε3/ε3
Aged CHD	ε3/ε3	950.4	NA	1373.5	866.4	NA	ε3/ε3
Aged CHD	ε3/ε3	1805.3	NA	1026.3	2071.1	NA	ε3/ε3
Aged CHD	ε3/ε3	830.3	NA	471.8	743.8	NA	ε3/ε3
Aged CHD	ε3/ε3	957.5	NA	1289.0	934.7	NA	ε3/ε3
Aged CHD	ε3/ε3	1651.8	NA	1801.6	1532.7	NA	ε3/ε3
Aged CHD	ε3/ε3	664.5	NA	1198.8	625.3	NA	ε3/ε3
Aged CHD	ε3/ε3	1199.7	NA	783.2	1284.2	NA	ε3/ε3
Aged CHD	ε3/ε3	1191.2	NA	640.4	1087.8	NA	ε3/ε3
Aged CHD	ε3/ε3	1929.1	NA	1883.8	1753.5	NA	ε3/ε3
Aged CHD	ε3/ε3	1515.2	NA	780.1	1407.5	NA	ε3/ε3
Aged CHD	ε3/ε3	883.8	NA	441.6	810.8	NA	ε3/ε3
Aged CHD	ε3/ε3	1568.4	NA	811.7	1411.8	NA	ε3/ε3
Aged CHD	ε3/ε3	865.3	NA	394.2	800.5	NA	ε3/ε3
Aged CHD	ε3/ε3	934.9	NA	564.9	877.3	NA	ε3/ε3
Aged CHD	ε3/ε3	905.2	NA	1169.2	763.6	NA	ε3/ε3

Aged CHD	ε3/ε3	773.1	NA	815.9	686.6	NA	ε3/ε3
Aged CHD	ε3/ε3	914.0	NA	515.0	864.9	NA	ε3/ε3
Aged CHD	ε3/ε3	853.4	NA	1144.4	801.0	NA	ε3/ε3
Aged CHD	ε3/ε3	707.5	NA	517.3	689.0	NA	ε3/ε3
Aged CHD	ε3/ε3	785.6	NA	495.2	725.5	NA	ε3/ε3
Aged CHD	ε3/ε3	1177.2	NA	866.9	1045.4	NA	ε3/ε3
Aged CHD	ε3/ε3	1771.9	NA	772.3	1926.6	NA	ε3/ε3
Aged CHD	ε3/ε3	920.1	NA	631.2	876.1	NA	ε3/ε3
Aged CHD	ε3/ε3	799.4	NA	666.9	792.6	NA	ε3/ε3
Aged CHD	ε3/ε3	1100.2	NA	739.5	1135.1	NA	ε3/ε3
Aged CHD	ε3/ε3	1704.3	NA	1659.9	1527.5	NA	ε3/ε3
Aged CHD	ε3/ε3	1148.9	NA	665.0	1058.8	NA	ε3/ε3
Aged CHD	ε3/ε3	771.1	NA	1143.7	691.4	NA	ε3/ε3
Aged CHD	ε3/ε3	1469.3	NA	1494.0	1260.5	NA	ε3/ε3
Aged CHD	ε3/ε3	1116.9	NA	577.3	1060.7	NA	ε3/ε3
Aged CHD	ε3/ε3	1145.2	NA	675.5	1116.1	NA	ε3/ε3
Aged CHD	ε3/ε3	1452.3	NA	828.4	1341.9	NA	ε3/ε3
Aged CHD	ε3/ε3	1056.3	NA	563.1	943.4	NA	ε3/ε3
Aged CHD	ε3/ε3	1673.1	NA	462.0	1756.9	NA	ε3/ε3
Aged CHD	ε3/ε3	1401.1	NA	761.2	1324.3	NA	ε3/ε3
Aged CHD	ε3/ε3	1146.8	NA	2112.3	1044.0	NA	ε3/ε3
Aged CHD	ε3/ε3	590.6	NA	377.4	579.4	NA	ε3/ε3
Aged CHD	ε3/ε3	1734.6	NA	1130.1	1521.0	NA	ε3/ε3
Aged CHD	ε3/ε3	1843.0	NA	901.7	1614.3	NA	ε3/ε3
Aged CHD	ε3/ε3	1308.3	NA	1556.8	1235.4	NA	ε3/ε3
Aged CHD	ε3/ε3	748.3	NA	371.1	638.8	NA	ε3/ε3
Aged CHD	ε3/ε3	979.1	NA	489.9	864.3	NA	ε3/ε3
Aged CHD	ε3/ε3	843.3	NA	391.8	807.9	NA	ε3/ε3
Aged CHD	ε3/ε3	746.7	NA	313.5	646.7	NA	ε3/ε3
Aged CHD	ε3/ε3	689.2	NA	835.3	583.3	NA	ε3/ε3
Aged CHD	ε3/ε3	1890.5	NA	758.6	1968.2	NA	ε3/ε3
Aged CHD	ε3/ε3	716.8	NA	650.9	705.1	NA	ε3/ε3
Aged CHD	ε3/ε3	904.0	NA	553.4	799.4	NA	ε3/ε3
Aged CHD	ε3/ε3	1012.2	NA	1155.4	983.2	NA	ε3/ε3
Aged CHD	ε3/ε3	1352.6	NA	713.8	1291.2	NA	ε3/ε3
Aged CHD	ε3/ε3	788.1	NA	446.2	771.5	NA	ε3/ε3
Aged CHD	ε3/ε3	962.2	NA	1703.7	941.3	NA	ε3/ε3
Aged CHD	ε3/ε3	625.6	NA	720.2	621.9	NA	ε3/ε3
Aged CHD	ε3/ε3	916.6	NA	859.7	935.4	NA	ε3/ε3
Aged CHD	ε3/ε3	1208.5	NA	624.5	1297.2	NA	ε3/ε3
Aged CHD	ε3/ε3	467.4	NA	359.1	483.1	NA	ε3/ε3
Aged CHD	ε3/ε3	739.5	NA	641.2	870.6	NA	ε3/ε3
Aged CHD	ε3/ε3	725.1	NA	626.6	733.5	NA	ε3/ε3
Aged CHD	ε3/ε3	1131.5	NA	639.1	1118.2	NA	ε3/ε3

Aged CHD	ε3/ε3	1099.5	NA	578.8	1201.2	NA	ε3/ε3
Aged CHD	ε3/ε3	1445.7	NA	838.3	1438.8	NA	ε3/ε3
Aged CHD	ε3/ε3	1764.9	NA	1757.6	1788.7	NA	ε3/ε3
Aged CHD	ε3/ε3	751.0	NA	370.7	703.1	NA	ε3/ε3
Aged CHD	ε3/ε3	1632.1	NA	619.5	1955.9	NA	ε3/ε3
Aged CHD	ε3/ε3	903.4	NA	533.6	871.8	NA	ε3/ε3
Aged CHD	ε3/ε3	720.9	NA	383.6	791.8	NA	ε3/ε3
Aged CHD	ε3/ε3	1492.4	NA	815.5	1605.1	NA	ε3/ε3
Aged CHD	ε3/ε3	1399.2	NA	1036.9	1488.3	NA	ε3/ε3
Aged CHD	ε3/ε3	840.8	NA	615.8	969.7	NA	ε3/ε3
Aged CHD	ε3/ε3	1016.8	NA	607.2	1021.8	NA	ε3/ε3
Aged CHD	ε3/ε3	1182.7	NA	894.4	1078.9	NA	ε3/ε3
Aged CHD	ε3/ε3	2523.9	NA	1225.4	2214.5	NA	ε3/ε3
Aged CHD	ε3/ε3	529.6	NA	277.7	497.0	NA	ε3/ε3
Aged CHD	ε3/ε3	947.2	NA	593.0	902.9	NA	ε3/ε3
Aged CHD	ε3/ε3	1083.2	NA	547.7	1015.3	NA	ε3/ε3
Aged CHD	ε3/ε3	416.5	NA	619.6	419.0	NA	ε3/ε3
Aged CHD	ε3/ε3	522.8	NA	220.5	580.8	NA	ε3/ε3
Aged CHD	ε3/ε3	931.4	NA	493.4	859.9	NA	ε3/ε3
Aged CHD	ε3/ε3	865.6	NA	503.1	961.6	NA	ε3/ε3
Aged CHD	ε3/ε3	1159.6	NA	633.8	1241.9	NA	ε3/ε3
Aged CHD	ε3/ε3	815.9	NA	376.1	784.9	NA	ε3/ε3
Aged CHD	ε3/ε3	1346.6	NA	906.2	1419.9	NA	ε3/ε3
Aged CHD	ε3/ε3	600.9	NA	436.7	548.4	NA	ε3/ε3
Aged CHD	ε3/ε3	1282.2	NA	743.1	1278.8	NA	ε3/ε3
Aged CHD	ε3/ε3	469.6	NA	202.4	498.6	NA	ε3/ε3
Aged CHD	ε3/ε3	718.0	NA	481.0	697.2	NA	ε3/ε3
Aged CHD	ε3/ε3	752.1	NA	1335.4	716.3	NA	ε3/ε3
Aged CHD	ε3/ε3	981.8	NA	586.6	1045.3	NA	ε3/ε3
Aged CHD	ε3/ε3	1140.3	NA	648.4	1045.5	NA	ε3/ε3
Aged CHD	ε3/ε3	650.4	NA	404.2	611.2	NA	ε3/ε3
Aged CHD	ε3/ε3	891.8	NA	456.1	903.6	NA	ε3/ε3
Aged CHD	ε3/ε3	1035.5	NA	615.5	1096.8	NA	ε3/ε3
Aged CHD	ε3/ε3	523.4	NA	351.6	590.2	NA	ε3/ε3
Aged CHD	ε3/ε3	1103.1	NA	635.0	1154.6	NA	ε3/ε3
Aged CHD	ε3/ε3	621.2	NA	388.2	601.7	NA	ε3/ε3
Aged CHD	ε3/ε3	862.2	NA	505.7	851.4	NA	ε3/ε3
Aged CHD	ε3/ε3	1485.3	NA	974.5	1615.6	NA	ε3/ε3
Aged CHD	ε3/ε3	966.2	NA	628.3	1037.3	NA	ε3/ε3
Aged CHD	ε3/ε3	1138.1	NA	571.1	1135.3	NA	ε3/ε3
Aged CHD	ε3/ε3	1925.2	NA	929.9	2086.2	NA	ε3/ε3
Aged CHD	ε3/ε3	662.3	NA	371.2	675.5	NA	ε3/ε3
Aged CHD	ε3/ε3	634.8	NA	341.9	681.3	NA	ε3/ε3
Aged CHD	ε3/ε3	1183.3	NA	770.6	1319.5	NA	ε3/ε3

Aged CHD	ε3/ε3	806.7	NA	453.8	850.1	NA	ε3/ε3
Aged CHD	ε3/ε3	1713.3	NA	903.9	1761.7	NA	ε3/ε3
Aged CHD	ε3/ε3	864.9	NA	543.7	1022.6	NA	ε3/ε3
Aged CHD	ε3/ε3	921.2	NA	548.0	854.3	NA	ε3/ε3
Aged CHD	ε3/ε3	1798.4	NA	839.3	1939.5	NA	ε3/ε3
Aged CHD	ε3/ε3	733.7	NA	384.7	742.7	NA	ε3/ε3
Aged CHD	ε3/ε3	853.3	NA	498.2	909.3	NA	ε3/ε3
Aged CHD	ε3/ε3	1202.7	NA	949.0	1133.1	NA	ε3/ε3
Aged CHD	ε3/ε3	1048.3	NA	452.5	1044.8	NA	ε3/ε3
Aged CHD	ε3/ε3	711.5	NA	890.3	691.8	NA	ε3/ε3
Aged CHD	ε3/ε3	619.8	NA	285.8	658.4	NA	ε3/ε3
Aged CHD	ε3/ε3	591.5	NA	817.6	620.1	NA	ε3/ε3
Aged CHD	ε3/ε3	729.9	NA	777.6	636.8	NA	ε3/ε3
Aged CHD	ε3/ε3	1098.8	NA	1002.1	1024.4	NA	ε3/ε3
Aged CHD	ε3/ε3	1060.9	NA	724.0	1019.6	NA	ε3/ε3
Aged CHD	ε3/ε3	1231.0	NA	1641.1	1196.1	NA	ε3/ε3
Aged CHD	ε3/ε3	1690.5	NA	881.2	1689.6	NA	ε3/ε3
Aged CHD	ε3/ε3	1390.0	NA	690.0	1481.1	NA	ε3/ε3
Aged CHD	ε3/ε3	866.9	NA	759.4	859.8	NA	ε3/ε3
Aged CHD	ε3/ε3	1067.5	NA	644.9	1023.0	NA	ε3/ε3
Aged CHD	ε3/ε3	667.6	NA	448.7	783.0	NA	ε3/ε3
Aged CHD	ε3/ε3	1475.6	NA	938.4	1487.1	NA	ε3/ε3
Aged CHD	ε3/ε3	878.5	NA	477.1	929.4	NA	ε3/ε3
Aged CHD	ε3/ε3	1313.0	NA	713.2	1360.3	NA	ε3/ε3
Aged CHD	ε3/ε3	921.3	NA	526.9	987.8	NA	ε3/ε3
Aged CHD	ε3/ε3	1112.5	NA	855.4	1057.6	NA	ε3/ε3
Aged CHD	ε3/ε3	651.9	NA	596.3	628.8	NA	ε3/ε3
Aged CHD	ε3/ε3	1233.7	NA	1153.7	1411.1	NA	ε3/ε3
Aged CHD	ε3/ε3	947.3	NA	767.2	920.9	NA	ε3/ε3
Aged CHD	ε3/ε3	844.1	NA	750.2	790.3	NA	ε3/ε3
Aged CHD	ε3/ε3	1214.0	NA	553.9	1314.0	NA	ε3/ε3
Aged CHD	ε3/ε3	760.8	NA	440.5	869.5	NA	ε3/ε3
Aged CHD	ε3/ε3	1886.0	NA	968.8	2007.9	NA	ε3/ε3
Aged CHD	ε3/ε3	1451.7	NA	607.0	1455.5	NA	ε3/ε3
Aged CHD	ε3/ε4	1012.2	NA	275.3	1075.1	269.0	ε3/ε4
Aged CHD	ε3/ε4	934.7	NA	808.8	869.1	195.9	ε3/ε4
Aged CHD	ε3/ε4	1375.2	NA	536.1	1450.1	400.4	ε3/ε4
Aged CHD	ε3/ε4	1716.5	NA	554.4	1633.5	527.4	ε3/ε4
Aged CHD	ε3/ε4	2178.1	NA	679.6	2132.6	512.7	ε3/ε4
Aged CHD	ε3/ε4	1178.8	NA	447.7	1362.9	331.3	ε3/ε4
Aged CHD	ε3/ε4	1365.5	NA	875.5	1242.1	330.1	ε3/ε4
Aged CHD	ε3/ε4	738.2	NA	194.8	822.5	232.0	ε3/ε4
Aged CHD	ε3/ε4	1718.4	NA	513.3	1899.6	567.9	ε3/ε4
Aged CHD	ε3/ε4	734.8	NA	654.4	622.6	224.5	ε3/ε4

Aged CHD	ε3/ε4	1272.7	NA	1064.1	1266.9	453.7	ε3/ε4
Aged CHD	ε3/ε4	1187.1	NA	927.5	1186.4	313.3	ε3/ε4
Aged CHD	ε3/ε4	682.1	NA	286.4	733.3	211.7	ε3/ε4
Aged CHD	ε3/ε4	735.5	NA	304.4	706.8	195.9	ε3/ε4
Aged CHD	ε3/ε4	1290.6	NA	650.6	1314.3	357.5	ε3/ε4
Aged CHD	ε3/ε4	867.7	NA	306.0	922.3	268.9	ε3/ε4
Aged CHD	ε3/ε4	1436.3	NA	519.0	1527.8	322.4	ε3/ε4
Aged CHD	ε3/ε4	1154.7	NA	394.9	1343.8	285.7	ε3/ε4
Aged CHD	ε3/ε4	924.7	NA	467.9	917.6	301.9	ε3/ε4
Aged CHD	ε3/ε4	835.1	NA	350.1	849.3	220.1	ε3/ε4
Aged CHD	ε3/ε4	381.1	NA	199.7	371.9	109.0	ε3/ε4
Aged CHD	ε3/ε4	893.8	NA	326.3	904.0	285.2	ε3/ε4
Aged CHD	ε3/ε4	1221.2	NA	442.7	1237.0	391.2	ε3/ε4
Aged CHD	ε3/ε4	808.2	NA	251.8	1007.0	248.4	ε3/ε4
Aged CHD	ε3/ε4	868.2	NA	483.7	952.4	161.4	ε3/ε4
Aged CHD	ε3/ε4	849.0	NA	609.5	794.0	260.6	ε3/ε4
Aged CHD	ε3/ε4	1211.4	NA	436.6	1164.4	382.9	ε3/ε4
Aged CHD	ε3/ε4	1200.3	NA	503.7	1327.4	357.9	ε3/ε4
Aged CHD	ε3/ε4	1901.2	NA	559.0	2181.8	576.1	ε3/ε4
Aged CHD	ε3/ε4	596.1	NA	304.7	646.3	165.2	ε3/ε4
Aged CHD	ε3/ε4	1087.6	NA	333.1	1266.4	337.0	ε3/ε4
Aged CHD	ε3/ε4	940.8	NA	323.6	1064.5	268.8	ε3/ε4
Aged CHD	ε3/ε4	1084.5	NA	533.8	1077.4	236.5	ε3/ε4
Aged CHD	ε3/ε4	1399.0	NA	349.7	1485.1	414.0	ε3/ε4
Aged CHD	ε3/ε4	1031.4	NA	356.3	1022.7	247.0	ε3/ε4
Aged CHD	ε3/ε4	608.6	NA	235.5	626.6	89.2	ε3/ε4
Aged CHD	ε3/ε4	844.3	NA	284.4	996.7	241.1	ε3/ε4
Aged CHD	ε3/ε4	1068.0	NA	1006.4	1074.7	245.8	ε3/ε4
Aged CHD	ε3/ε4	753.1	NA	255.2	797.1	210.4	ε3/ε4
Aged CHD	ε3/ε4	538.8	NA	303.7	536.7	155.5	ε3/ε4
Aged CHD	ε3/ε4	1229.8	NA	473.3	1120.5	301.6	ε3/ε4
Aged CHD	ε3/ε4	1594.1	NA	496.4	1527.4	460.2	ε3/ε4
Aged CHD	ε4/ε4	1032.6	NA	N/A	1052.3	776.5	ε4/ε4
Aged CHD	ε4/ε4	995.7	NA	N/A	927.1	777.4	ε4/ε4
Aged CHD	ε4/ε4	597.7	NA	N/A	577.1	509.0	ε4/ε4

Supplementary Table S4 Baseline characteristics of all young and aged CHD patients in dataset 1.

	Young CHD (n=75)	Aged CHD (n=218)	p value
Sex, man (%)	63 (84)	141 (64.7)	0.017
Age-years	37.4 ± 5.7	64.2 ± 10.6	0.000
Median (IQR)	38 (35-42)	65 (55-72)	
**Disease history (n, %)**			
Hypertension	21 (28)	141 (64.7)	<0.001
Dyslipidemia	33 (44)	60 (27.5)	0.008
Stroke	4 (5.3)	71 (32.6)	<0.001
Diabetes	3 (4)	35 (16.1)	0.008
**Clinical Biochemistry**			
TC (mmol/L)	4.9 ± 1.4	4.4 ± 1.2	0.019
TG (mmol/L)	2.2 ± 1.4	1.6 ± 0.9	0.001
HDL-C (mmol/L)	1.2 ± 0.3	1.2 ± 0.3	0.575
LDL-C (mmol/L)	3.2 ± 1.0	2.8 ± 0.9	0.017
ALT (U/L)	40 ± 52.3	20.3 ± 12.1	<0.001
Glucose (mmol/L)	5.5 ± 2	6.4 ± 2.2	0.002
Creatinine (μmol/L)	74.9 ± 15.9	79.5 ± 46.9	0.410
eGFR (ml/min/1.73m^2)	110.7 ± 17.2	88.9 ± 22.5	<0.001
**APOE Group**			
APOE2 (n, %)	10 (13.3)	32 (14.7)	0.265
APOE3 (n, %)	48 (64)	141 (64.7)	0.999
APOE4 (n, %)	17 (22.7)	45 (20.6)	0.543
**Medication**			
Statin (n, %)	4 (5.3)	46 (21.1)	<0.001

Values for continuous and categorical variables are expressed as median [25th; 75th percentile] and percentage, or mean ± standard deviation, respectively. The Kruskal–Walli's test for continuous variables and the chi-square test for categorical variables were used to determine significant difference between groups.

Note: Participants were grouped into three categories due to the small sample sizes of ε2/ε2, ε2/ε4, and ε4/ε4 genotypes: APOE2 (including ε2/ε2 and ε2/ε3 genotype), APOE3 (ε3/ε3 genotype), and APOE4 (including ε2/ε4, ε3/ε4 and ε4/ε4 genotype).

Keys: IQR, interquartile range; CHD, coronary heart disease; HDL, High density lipoprotein; LDL, Low density lipoprotein; TC, total cholesterol; TG, triglyceride; ALT, Alanine Transaminase; eGFR, estimated Glomerular Filtration Rate.

Supplementary Table S5 Concentration of apolipoproteins in all young and aged CHD patients in dataset 1.

ConC (ng/ml)	Young CHD (n=75)	Aged CHD (n=218)	P value
ApoA1	37845.9 ± 8556.6	39465.4 ± 10735.7	0.238
ApoA2	20543.9 ± 4731.9	18411.1 ± 4847.4	**0.001**
ApoA4	1180.9 ± 383.6	1275.8 ± 544.4	0.164
ApoB	1415.1 ± 479.9	1219.1 ± 462.2	**0.002**
ApoC1	1756.2 ± 412	1699.1 ± 561.7	0.419
ApoC2	3808.3 ± 1745.2	2808.8 ± 1319.9	**0.000**
ApoC3	6497.5 ± 2492.1	5645.5 ± 2690.3	**0.017**
ApoC4	34.2 ± 17.3	33.1 ± 23.1	0.698
ApoD	1307.2 ± 418.6	1272.9 ± 444.9	0.560
ApoE	1357.2 ± 696.2	1185.7 ± 562.4	**0.034**
ApoH	2429.3 ± 536.9	2288.1 ± 623.6	0.081
ApoJ	4001 ± 1013.3	3567.3 ± 986.1	**0.001**
ApoL1	270.3 ± 79.2	204.4 ± 72.6	**<0.001**
ApoM	484.2 ± 157.5	401.5 ± 149.4	**<0.001**
Lp(a)-KR	832.1 ± 858.2	1194.3 ± 1362.8	**0.033**
Lp(a)-CR	20.9 ± 22.6	34.3 ± 47.4	**0.019**

Values are expressed as mean standard deviation, respectively.

Keys: ConC: concentration of each peptide.

Supplementary Table S6. Demographic and clinical characteristics of the CHD subjects from UK Biobank according to age.

	Age < 45 years			Age >45 years		
	**Control (n=4597)**	**CHD (n=148)**	**p value**	**Control (n=23059)**	**CHD (n=3673)**	**p value**
**Demographic characteristics**						
Age (years)	43 (41, 44)	44 (42, 45)	<0.001	58 (52, 63)	62 (57, 66)	<0.001
Sex (Male, %)	2234 (48.6)	97 (65.5)	<0.001	9665 (41.9)	2304 (62.7)	<0.001
Ethnic (Caucasian, %)	3388 (73.7)	106 (71.6)	0.572	19181 (83.2)	3095 (84.3)	0.102
Ever smoked (n, %)	2494 (54.3)	94 (63.5)	0.025	13246 (57.4)	2472 (67.3)	<0.001
BMI (Kg/m^2)	26.1 (23.5, 29.2)	28.7 (25.3, 31.9)	<0.001	26.6 (24.0, 29.6)	28.2 (25.6, 31.6)	<0.001
SBP (mmHg)	128 (118, 140)	132 (122.8, 145.3)	0.002	139 (126, 152)	142 (130, 156)	<0.001
**Clinical Biochemistry**						
TC (mmol/L)	5.3 (4.7, 6.0)	5.4 (4.7, 6.3)	0.536	5.7 (5.0, 6.5)	5.2 (4.3, 6.2)	<0.001
TG (mmol/L)	1.3 (0.9, 1.9)	1.8 (1.1, 2.5)	<0.001	1.5 (1.0, 2.1)	1.7 (1.2, 2.4)	<0.001
HDL (mmol/L)	1.3 (1.1, 1.6)	1.2 (1.0, 1.3)	<0.001	1.4 (1.2, 1.7)	1.2 (1.0, 1.5)	<0.001
LDL (mmol/L)	3.3 (2.8, 3.9)	3.5 (2.8, 4.0)	0.167	3.6 (3.0, 4.2)	3.2 (2.5, 3.9)	<0.001
Lipoprotein(a) (mmol/L)	20.9 (9.2, 71)	25.2 (9.4, 80.5)	0.703	21.4 (9.6, 61)	24.2 (10.1, 73.1)	<0.001
ALT (U/L)	19.2 (14.1, 28.2)	23.1 (17, 33.5)	0.001	19.8 (15.4, 26.7)	22 (16.8, 30.2)	<0.001
Glucose (mmol/L)	4.8 (4.5, 5.1)	4.8 (4.5, 5.2)	0.202	4.9 (4.6, 5.3)	5 (4.7, 5.5)	<0.001
Creatinine (umol/L)	69.9 (61.1, 80)	71.6 (63.5, 84.9)	0.004	69.8 (60.8, 80)	75.2 (65, 86.5)	<0.001
**Medication**						
Anti-cholesterol (n, %)	117 (2.5)	26 (17.6)	<0.001	1815 (7.9)	1292 (35.2)	<0.001
Anti-blood pressure (n, %)	116 (2.5)	25 (16.9)	<0.001	2040 (8.8)	1186 (32.3)	<0.001
Anti-diabetes (n, %)	26 (0.6)	3 (2)	0.024	110 (0.5)	87 (2.4)	<0.001
**Olink data**			**p_{adj} value***			**p_{adj} value***
APOL1 (NPX)	0.1 (-0.2, 0.4)	0.02 (-0.25, 0.49)	0.408	0 (-0.3, 0.3)	-0.09 (-0.38, 0.21)	<0.001

Values for continuous and categorical variables are expressed as median [25th; 75th percentile] and percentage, respectively. The Kruskal–Wallis test for continuous variables and the chi-square test for categorical variables was used to determine significant difference between groups. *: Multinomial logistic regression was further performed to adjust for all covariates presented in the tables, including sex, BMI, clinical biochemistry parameters, and medication. Abbreviations: BMI, Body mass index; HDL, High density lipoprotein; LDL, Low density lipoprotein; ALT, alanine aminotransferase; NPX, Normalized Protein expression; SBP, systolic blood pressure; TC, total cholesterol; TG, triglycerides.

References

1. Begcevic Brkovic I, Zohrer B, Scholz M, et al. Simultaneous Mass Spectrometry-Based Apolipoprotein Profiling and Apolipoprotein E Phenotyping in Patients with ASCVD and Mild Cognitive Impairment. Nutrients. 2022;14(12).
2. Ceglarek U, Dittrich J, Becker S, Baumann F, Kortz L, Thiery J. Quantification of seven apolipoproteins in human plasma by proteotypic peptides using fast LC-MS/MS. Proteomics Clinical applications. 2013;7(11-12):794-801.
3. Toth CA, Kuklenyik Z, Jones JI, et al. On-column trypsin digestion coupled with LC-MS/MS for quantification of apolipoproteins. Journal of proteomics. 2017;150:258-267.
4. Zhang Y, Ren X, Zhou Z, et al. Simultaneous quantitative LC-MS/MS analysis of 13 apolipoproteins and lipoprotein (a) in human plasma. The Analyst. 2024;149(12):3444-3455.
